# The Photochemistry of Amino Acids Produced on the Polar Cryovolcanic Regions of Titan

**DOI:** 10.1021/acsearthspacechem.4c00376

**Published:** 2025-03-08

**Authors:** Diogo Gonçalves, Florence Hofmann, Severin Wipf, Riccardo Giovanni Urso, Jana Bocková, Cornelia Meinert, Paul Brandon Rimmer, Gautam Dutta Stroscio, Nir Goldman, Andreas Elsaesser, Bruno Pedras, Zita Martins

**Affiliations:** † Centro de Química Estrutural, Institute of Molecular Sciences and Department of Chemical Engineering, Instituto Superior Técnico, 37809 Universidade de Lisboa, Av. Rovisco Pais 1, 1049-001 Lisbon, Portugal; ‡ Institute for Bioengineering and Biosciences and Department of Chemical Engineering, Instituto Superior Técnico, Universidade de Lisboa, Av. Rovisco Pais 1, 1049-001 Lisbon, Portugal; § Freie Universität Berlin, Department of Physics, Experimental Biophysics and Space Science, Arnimallee 14, 14195 Berlin, Germany; ∥ 53294 INAF-Osservatorio Astrofisico di Catania, Via Santa Sofia 78, 95123 Catania, Italy; ⊥ 439710 Université Côte d’Azur-CNRS, ICN, UMR7272, 06108 Nice, France; # Cavendish Laboratory, 2152 University of Cambridge, JJ Thomson Ave, CB3 0HE Cambridge, U.K.; ∇ 4578 Lawrence Livermore National Laboratory, Livermore, California 94550, United States; ○ Department of Chemical Engineering, University of California, Davis, California 95616, United States; ◆ Associate Laboratory i4HB−Institute for Health and Bioeconomy, Instituto Superior Técnico, 386370 Universidade de Lisboa, Av. Rovisco Pais 1, 1049-001 Lisbon, Portugal

**Keywords:** bubble bursting, Hadley cells, meridional winds, Dragonfly, solar irradiation, alanine, glycine

## Abstract

The cryovolcanic regions of Titan offer transient opportunities for prebiotic molecules to exist in water–ammonia solutions on the surface of the Saturnian moon. The upcoming NASA’s Dragonfly mission will search for high nitrogen concentrations and amino acids on Titan’s equatorial terrains. Cryovolcanic features, however, are most common on the polar regions. To mitigate the distance, bubble bursting may encapsulate the prebiotic molecules into aerosols, which Titan’s Hadley circulation would subsequently transport to the equator. We investigate whether alanine and glycine survive this meridional journey. Despite the unconstrained meridional wind velocities, our results suggest that the amino acids can survive the transport through the mesosphere. Dragonfly may find cryo-volcanogenic amino acids on Titan’s equator. Further, the interaction between the two amino acids increased 10-fold the photodegradation rate of glycine. We justify it based on changes in the environment polarity.

## Graphical abstract

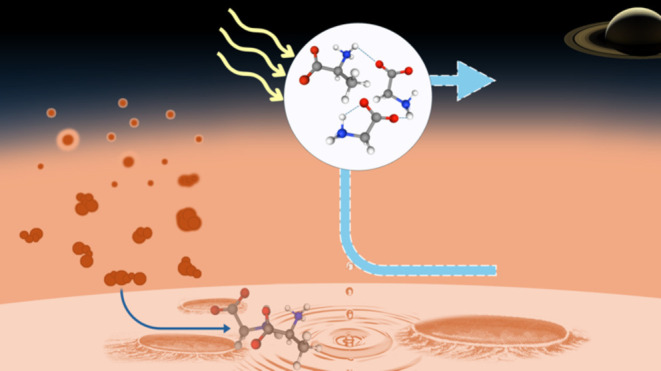

## 1 Introduction

On the surface of Titan, Saturn’s largest moon, liquid water may temporarily exist after some of the subsurface water ocean is extruded through the icy crust by cryovolcanic events. Following tentative evidence provided by the Cassini mission, the existence of cryovolcanism on this moon has remained unsettled, with the debate still active regarding the nature, origin, and geological dispersion of the cryovolcanic features on the surface of the moon.^1,2^ Despite the uncertainty on the exact composition of the cryolava, it is assumed that it ultimately originates from the subsurface water–ammonia ocean.^3^ Crucially, laboratory work has shown that a water–ammonia cryolava could hydrolyze Titan’s aerosols to form prebiotic molecules, such as the amino acids glycine and alanine.^2,4–7^ This raises the possibility that cryovolcanic water-based melts produce and interact with prebiotic molecules on Titan’s surface.

Recently, Wood and Radebaugh argued in favor of cryovolcanism on the surface of Titan while presenting geomorphological evidence that the polar regions are enriched in cryovolcanic formations.^8^ The higher incidence in the polar regions is explained by the thin and low-elevation polar crusts.^8,9^ The high latitudes at which cryovolcanic regions may harbor prebiotic chemistry contrast with the low-latitude landing location of NASA’s Dragonfly mission, whose science goals include the search for high nitrogen and amino acid concentrations on Titan’s surface.^10^ Herein, we investigate whether the prebiotic molecules produced on the polar regions could be measured by the Dragonfly lander.

### 1.1 Bubble Bursting and Hadley Circulation

In accordance with recent suggestions by Cordier et al.,^11^ bubble bursting can propel prebiotic molecules into the atmosphere. Gases dissolved in the cryolava flows—mostly methane (CH_4_), carbon dioxide (CO_2_), and ammonia (NH_3_)^12^—may coalesce to produce bubbles. After floating to the free surface of the cryolava flows, the gas bubbles shall break and thrust into the atmosphere tiny film droplets followed by larger jet drops.^11,13,14^ The drops dispersed into the atmosphere are expected to encapsulate the organic molecules that were dissolved in the cryolava flows or deposited on its surface. If true, the aerosolized organic matter should include the prebiotic molecules produced from the hydrolysis of Titan’s atmospheric macromolecules. Similar capillary processes may have formed the organic-rich grains from Enceladus’ plumes detected by the Cassini mission.^15,16^ The cryovolcanic droplets produced on Titan should quickly freeze and, facilitated by the low gravity and aeolian processes,^17^ be transported over extensive distances as atmospheric aerosols.

One possible transportation mechanism is Titan’s meridional circulation.^18^ The unequal hemispherical irradiation, brought about by Titan’s 26.7° obliquity with respect to the Sun, drives a single pole-to-pole atmospheric cell with upwelling at the summer pole and subsidence at the winter pole. The pole-to-pole cells last about half a Titan-yr, reversing their direction during Titan’s equinoxes. Flowing between the two poles, meridional (across latitudes) winds may seed the icy aerosols from the summer pole to lower latitudes. They are the transportation mechanism by which prebiotic molecules, encapsulated in aerosols produced by bubble bursting,^11^ may reach the equatorial regions and be sampled by Dragonfly.

Unfortunately, the summer pole upwelling reaches altitudes higher than 600 km,^19^ well into Titan’s mesosphere. Prebiotic molecules transported by it would thus be subject to energetic irradiation. Although the top branch of the meridional circulation is not high enough for energetic photoelectrons, electrons from the Saturnian magnetosphere, energetic ions, or galactic cosmic rays to reach the organic molecules,^20^ the latter should nevertheless be exposed to most solar photons that reach Titan’s atmosphere. This exposure is accentuated by the depletion of organic haze in the upper-atmosphere of the summer pole, promoted by the same Hadley circulation.^19,21–24^ The summer pole thus emerges as the latitude of Titan where maximum insolation^25,26^ meets the least shielding from solar ultraviolet irradiation. In this work we investigate whether alanine and glycine embedded in water–ammonia aerosols can survive the irradiation dose expected at Titan’s summer pole mesosphere. We discuss the possibility of Dragonfly finding, on the equatorial regions of Titan, prebiotic molecules sourced from the poles.

To calculate the half-life of alanine and glycine in the mesosphere, we investigated their photodegradation in a simulated water–ammonia ice environment, as that expected in the cryovolcanic aerosols. We adapted an experimental concept extensively used to study the photochemistry of organic molecules (e.g., alanine and glycine)^27–33^ under space conditions.^34–40^ We irradiated nanolayers of glycine and alanine through thin water–ammonia ice layers deposited on them (Section 3.1). We similarly irradiated nanolayers with a 1:1 mixture of alanine and glycine, to understand if their close interaction in the icy aerosols would influence their individual photodegradation behaviors (Section 3.2). Through computational methods, we put forward an explanation for the increased photodegradation rate of glycine in the presence of alanine (Sections 3.3 and 3.4). Finally, we extrapolate the irradiation results to Titan conditions, using the modeled solar radiation flux expected in the summer pole mesosphere of Titan (Section 3.5).

## 2 Material and Methods

### 2.1 Sample Preparation

The amino acid nanolayers were deposited by vacuum sublimation as described by Gonçalves et al.^41^ For each sample, 150 mg of glycine (Sigma-Aldrich, 99.7% purity), racemic alanine (dl-alanine, TCI, 98.5% purity), or their mixture was added to a polytetrafluoroethylene (PTFE) crucible. The crucible was then heated at constant temperatures—110 °C (glycine) and 115 °C (alanine and alanine+glycine)—keeping the pressure in the chamber at 10^–6^–10^–5^ mbar. The deposition process was monitored using a quartz crystal microbalance, targeting thicknesses close to, but lower than, 100 nm forming at slow deposition rates (1–2 Å s^–1^). The amino acids were deposited onto calcium fluoride (CaF_2_) windows (20 mm diameter, 2 mm thick, Korth Kristalle GmbH) solvent- and plasma-cleaned before usage. To obtain the desired 1:1 molar ratio in the alanine+glycine mixture nanolayers, thoroughly mixed 122 mg of dl-alanine and 28 mg of glycine were added to the crucible. This ratio was validated by X-ray photoelectron spectroscopy (XPS) and kept consistent in all irradiated alanine+glycine samples (SD = 9.5%, *n* = 9), as monitored by the IR band intensities assigned to the δ­(CH), 1308 cm^–1^, and ω­(CH_2_), 1330 cm^–1^, vibrational modes—specific to the α-carbons of alanine and glycine, respectively (Table S1). To minimize the water content in the amino acid nanolayers, prior to the amino acids’ deposition, the crucible was heated for 15 min at 80 °C, while the windows were masked through a mobile shutter. To prevent organic contamination, the interior surfaces of the vacuum chamber were coated with regularly renewed aluminum foil.

The amino acid nanolayers were transferred at ambient conditions to an ultrahigh vacuum (UHV) chamber (*P* ≤ 10^–8^ mbar) hosting a sample holder in thermal contact with the coldfinger of a closed-cycle helium cryocooler (ARS). Each sample was fitted to the sample holder and kept at a 10^–8^ mbar vacuum overnight. It was then cooled down to 90 K at a 3 K min^–1^ rate. At 90 K, a mixture of 95% water vapor and 5% anhydrous ammonia gas (99.999%, Linde GmbH) was introduced into the vacuum chamber, forming a water–ammonia ice layer on top of the amino acid nanolayer (Figure 1a). The produced ice layers are characterized in Supporting Information (SI). Prior to the introduction in the UHV chamber, the mixture was prepared in a mixing chamber that allowed us to measure the partial vapor pressures of the two species. During the admission of the mixed gases to the UHV chamber, the total pressure was kept below 3.0 × 10^–6^ mbar, and the thickness of the ice layer was continuously monitored at the ν_1_ water vibrational mode (Figure 1b). A hollow cylinder at the back of the sample holder inhibited condensation on both sides of the sample substrate, ensuring that the ice layer formed exclusively on top of the amino acid nanolayer (Figure 1c). The mixing ratio was chosen based on recent experiments on the water–ammonia hydrolysis of organic aerosol analogues^2^ and recent estimations of the composition of Titan’s ocean.^42^ The 90 K operating temperature was chosen not only for its similarity to Titan’s average surface temperature—94 K—but also for being lower than the ammonia sublimation temperature at UHV pressures—also 94 K.^43,44^

**Figure 1:**
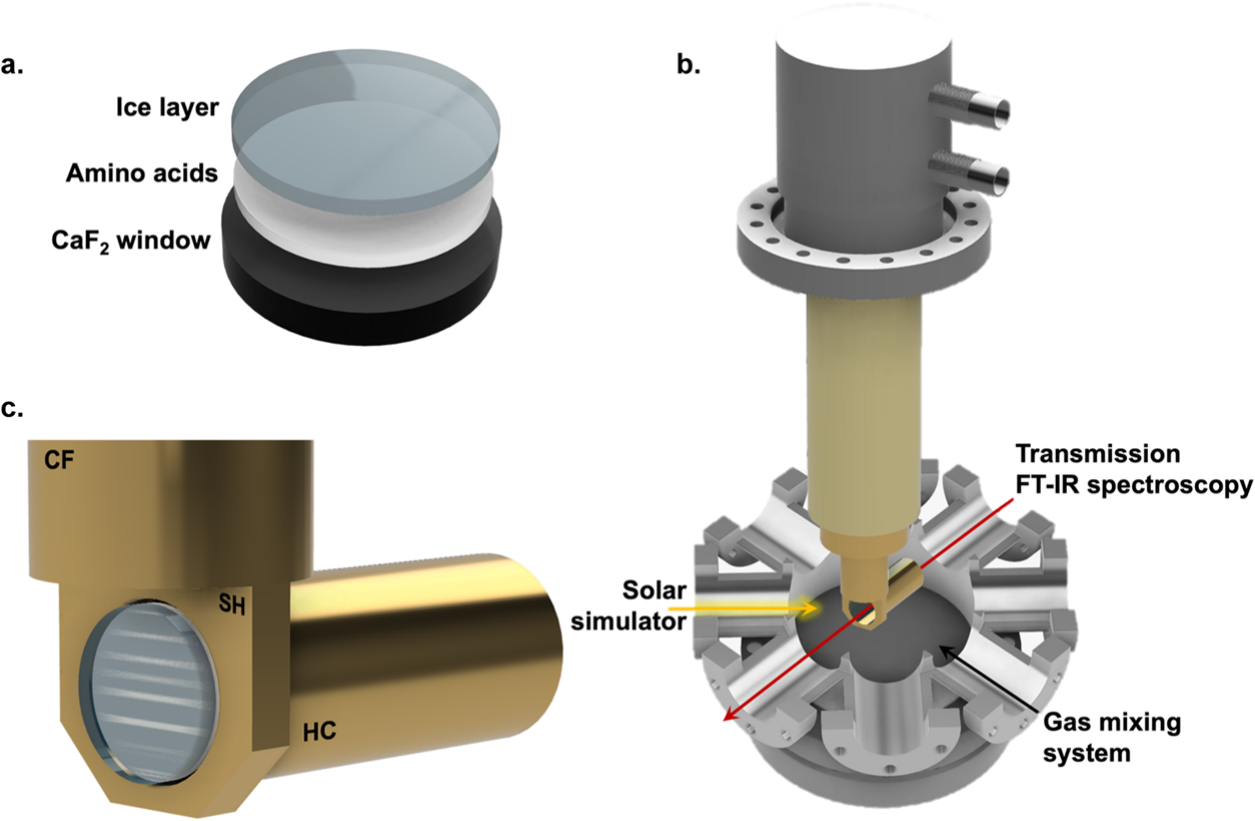
Layered sample structure and irradiation setup. **a.** Each irradiated sample (layer thicknesses not to scale) was built in two steps: first, an amino acid nanolayer was deposited onto a CaF_2_ window at room temperature through vacuum sublimation; second, a water–ammonia ice layer was grown on top of it at 90 K. **b.** Schematic view of the UHV chamber which enables simultaneous UV irradiation, gas admission, and *in situ* monitorization through transmission FT-IR spectroscopy. **c.** Detail of the coldfinger (CF), sample holder (SH), and hollow cylinder (HC) in the UHV chamber, and a sample in its position within the setup (sample layer thicknesses not to scale).

### 2.2 Transmission FT-IR Spectroscopy

After the vacuum deposition of the amino acids, each produced nanolayer was analyzed by transmission Fourier-Transform infrared (FT-IR) spectroscopy, at room temperature, inside a controlled nitrogen environment (Glovebox, MBraun), using an ArcOptix OEM FT-IR spectrometer (5000–832 cm^–1^) featuring an internal light source (SiC globar) and a MCT (4-TE cooled) detector. Spectra were recorded at a 4 cm^–1^ resolution averaged over 20 scans. The ice deposition and irradiation experiments were monitored through an ArcOptix OEM FT-IR spectrometer (5000–834 cm^–1^) fitted to the UHV chamber (Figure 1b), which afforded us *in situ* analysis. The IR beam passes through zinc selenide (ZnSe) windows installed on the UHV chamber and arrives at normal incidence with respect to the sample surface. Both the UHV chamber and the FT-IR spectrometer are hosted in a nitrogen-purged environment to reduce atmospheric contributions. Spectra were acquired with a 0.5 cm^–1^ resolution and 30 scans. For the analysis of the data here reported, all spectra were smoothed using a Savitzky-Golay method with window size 40 and polynomial order 2.

### 2.3 Sample Irradiation

The layered samples were exposed to continuous UV/vis/NIR irradiation provided by a 300 W solar simulator (Sciencetech) that we used to simulate the energetic processing of organic molecules on Titan’s summer pole. All irradiations lasted 3 h, with samples being exposed to the light of a standard xenon short arc lamp (Ushio, UXL-302-O), with a total photon flux between 200 and 250 nm of *F*_200–250 nm_ = (52.4 ± 1.5) × 10^13^ photon cm^–2^ s^–1^ (Figure S3). Complementary experiments under UV-enhanced conditions were conducted using a mercury-enhanced xenon short arc lamp (Hamamatsu, L2483), with a total photon flux between 200 and 250 nm *F*_200–250 nm_ = (36.8 ± 1.1) × 10^14^ photon cm^–2^ s^–1^ (Figure S3), measured with a calibrated UV/vis spectrometer (Ocean Optics). To constrain the role of the ice layer, we further irradiated the alanine and alanine+glycine samples in its absence. Seven control assays—where we did not employ any irradiation—were also performed, one with a glycine nanolayer, three with alanine nanolayers, and three with alanine+glycine nanolayers. These experiments demonstrated the sample stability during the 3 h in the absence of irradiation. Each irradiation experiment, listed in Table S2, was repeated three times.

### 2.4 Determination of the Kinetic Degradation Parameters

To distinguish the individual degradation rates of alanine and glycine in the alanine+glycine mixture samples the kinetic evolution of each amino acid was measured at IR bands specific for their α-carbons, that in the mixture are found at 1308 cm^–1^ for alanine and at 1330 cm^–1^ for glycine (Figure 2). Assuming that the number of molecules is proportional to the absorption band area *A_t_* at irradiation time *t*, for each time point, the relative abundance of a given amino acid (*N*(*t*)/*N*_0_) was calculated from the number of degraded molecules (*N*_deg_) according to eq 1.

1 $$\frac{N\left(t\right)}{N_{0}}=\frac{N_{0}-N_{\deg}\left(t\right)}{N_{0}}=\frac{A_{0}+A_{t}^{\deg}}{A_{0}}$$

with *A*_*t*_^deg^ being the integration of the (negative) absorption band quantifying the amino acids degraded during the irradiation experiment up to time *t*, and *A*_0_ the integration of the same absorption band at the start of the irradiation quantifying the initial amino acid abundance. *A*_*t*_^deg^ was calculated by *A*_*t*_^deg^ = −ln­(*I*(*t*)/*I*_0_), with *I*_0_ being the intensity at the start of the irradiation and *I_t_* the intensity after time *t* of irradiation. *A*_0_ was calculated from *A*_0_ = −ln­(*I*_0_/*I*_empty_), with *I*_empty_ being the reference spectrum obtained from an empty CaF_2_ window at the same experimental conditions, coated with the water–ammonia ice layer.

**Figure 2:**
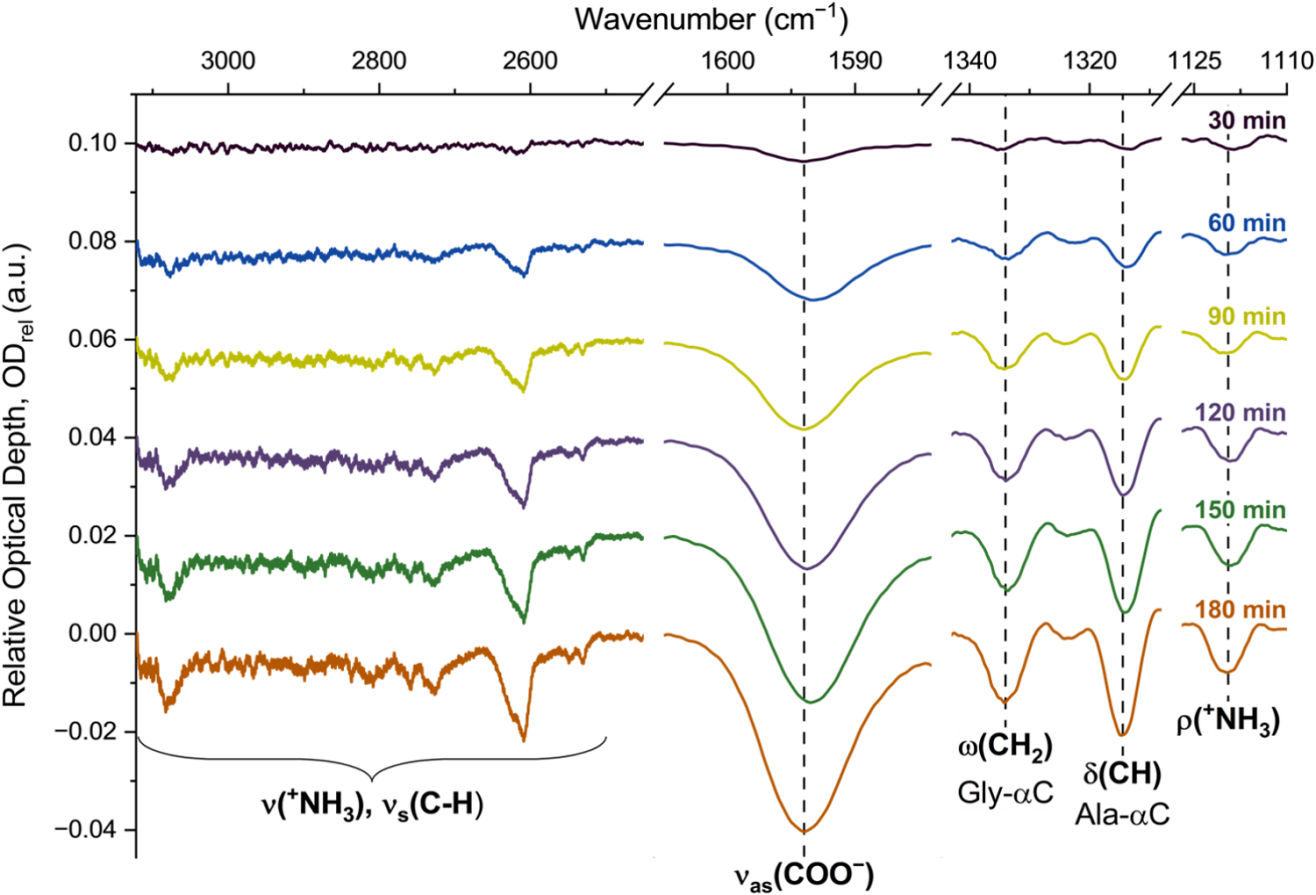
By evaluating the glycine-specific ω­(CH_2_) and the alanine-specific δ­(CH) modes in the alanine+glycine sample, we assess the evolution of the individual amino acids during irradiation. The absorption spectra were calculated relative to the intensity (*I*) collected at the beginning of the irradiation: OD_rel_ = ln­(*I_t_*/*I*_*t*=0_). Negative absorption signals the destruction of the amino acids during irradiation.

Based on the transmission spectra of glycine thin layers three times thicker than ours,^27^ we have assumed that our amino acid nanolayers are optically thin within our irradiation spectrum. This implies that all amino acids are irradiated by the same photon flux and that their photodegradation follows a first-order kinetics given by *N*(*t*) = *N*_0_ × e^–Jt^, with *J* being the degradation rate. *J* was calculated from the slope of the linear regression calculated from ln­(*N*(*t*)/*N*_0_) plotted against irradiation time *t*. For this purpose, the 12 data points collected every 15 min since *t* = 15 min to *t* = 180 min were considered. The relative abundance at each time point was averaged over the triplicate experiments, with the standard error for each time point accounted for in the linear regression. The degradation rate is presented as *J* = −slope ± SE × *t*(0.05, *n* – 2), *n* = 12, with SE being the standard error of the slope, as computed with the “Fit Linear with X Error” function from OriginPro, Version 2024 (OriginLab Corporation, Northampton, MA, USA), and *t*(0.05, 10) = 2.228 being from a Student’s *t*-distribution with a 95% confidence interval and *n* – 2 = 10 degrees of freedom. In the calculation of the trendlines and respective *R* ^2^ values of the time plots in Figures 3 and 4, we have also considered the *t* = 0 time point, with *N*(*t* = 0)/*N*_0_ = 1. The cross sections of destruction were computed through σ_des_ = *J*/*F*, from the photon flux (*F*) employed in the irradiation experiments. Based on the experimentally determined σ_des_ and the modeled Titan photon fluxes (*F*) we extrapolate the degradation rate (*J*) and respective half-lives—*t*_1/2_ = ln­(2/*J*)—to Titan’s summer pole. In these calculations, we considered the photon fluxes within the 200–250 nm range (*F*_200–250 nm_), which is likely the most relevant interval for the photodegradation of the two amino acids.^27,31,32^

**Figure 3:**
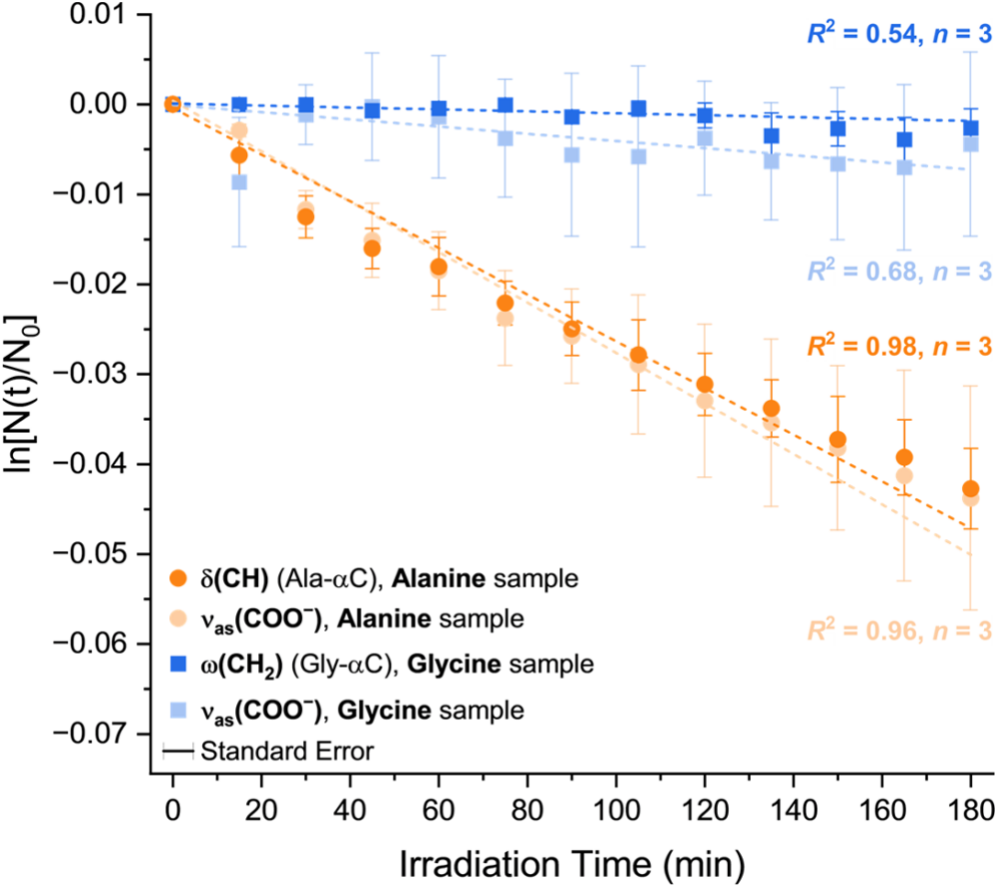
Time evolution of the relative abundance of pure glycine and pure alanine during irradiation. The negligible degradation of glycine, *J*_CH_2__^gly smp^ = (1.57 ± 1.38) × 10^–5^ min^–1^, contrasts sharply with the significant degradation of alanine, *J*_CH_^ala smp^ = (21.51 ± 4.02) × 10^–5^ min^–1^.

**Figure 4:**
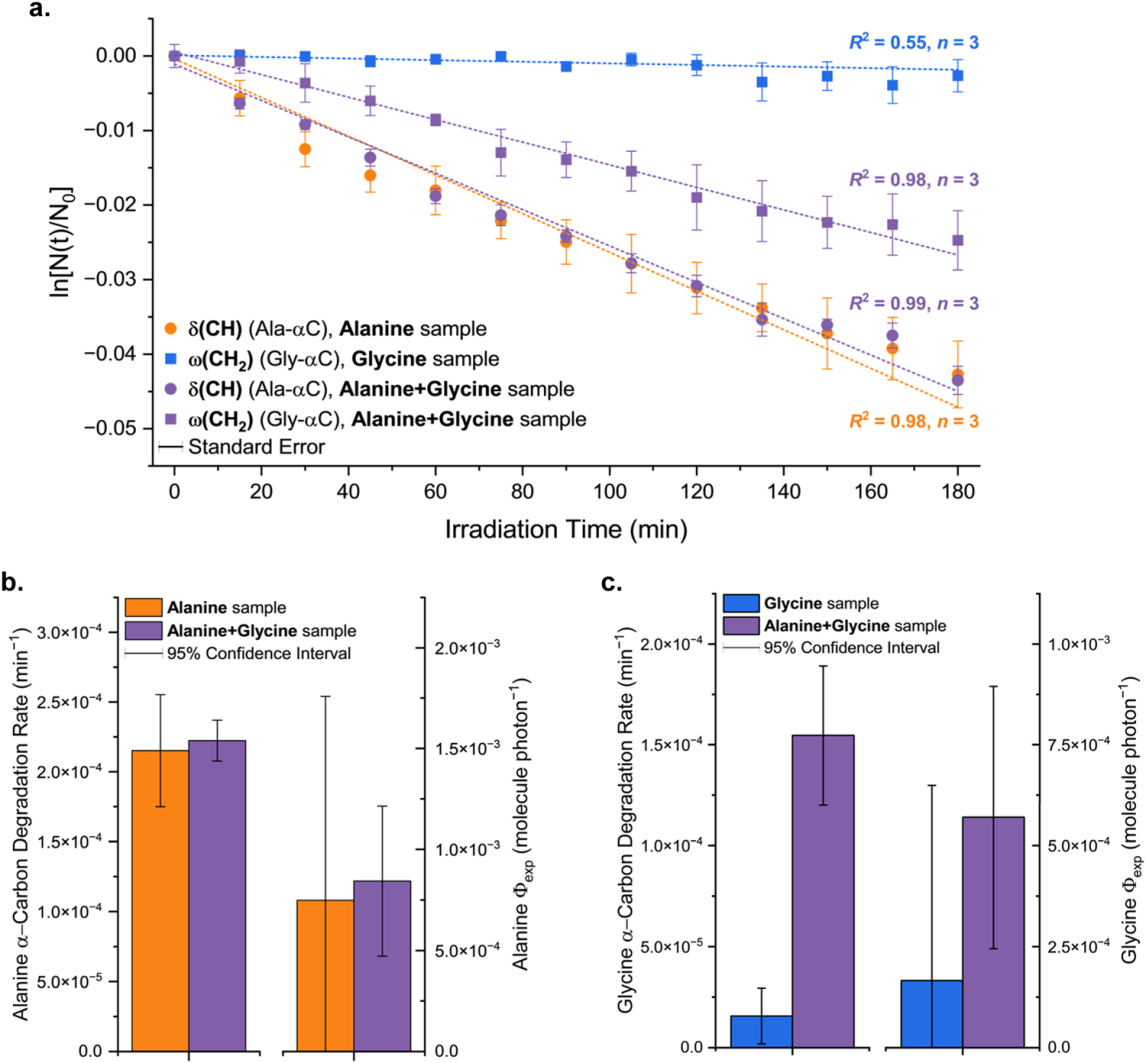
Effect of alanine and glycine on the photodegradation behavior of the other. **a.** In both pure and mixture samples, alanine degrades at the same rate. On the other hand, the degradation of glycine in the presence of alanine (alanine+glycine sample) differs significantly from the negligible degradation measured in the glycine sample. **b.** Although the alanine photodegradation rate remained unchanged (left), the errors associated with the quantum efficiency of photodegradation of alanine in both samples (right) were too large to assert their similarity. **c.** The presence of alanine increased the degradation rate of glycine 10-fold (left). After accounting for the incident photon flux and the initial number of molecules, the quantum efficiency of degradation (Φ_exp_) of glycine in the alanine+glycine sample was also statistically higher than in the pure glycine sample (right).

#### 2.4.1 Quantum Efficiency of Photodegradation

Poch et al. reported a strong dependence of the photolysis rate on the thickness of the irradiated organic layer, even at the scale of tenths of nanometers we have herein tested.^32^ The authors showed that the experimental quantum efficiency of photodegradation (Φ_exp_)—the number of degraded molecules per incident photon, Φ_exp_ = *N*_deg_/*N*_incident. photons_—accounts for the degradation extent independently of the layer thickness. We have adopted this methodology, given that the number of alanine and glycine molecules in the alanine+glycine samples differs substantially from that deposited in the pure alanine and pure glycine samples. As Poch et al.,^32^ we have calculated the number of degraded molecules through eq 2:

2 $$N_{\deg}=N_{0}\times \frac{N_{\deg}}{N_{0}}=N_{0}\times \frac{A_{\deg}}{A_{0}}$$

and the number of incident photons through eq 3:

3 $$N_{\mathrm{incident photons}}=F_{200-250\mathrm{nm}}\times \pi \times r^{2}\times \cos\left(45^{{}^{\circ}}\right)\times \Delta t$$

where *r* = 9 mm is the sample radius, placed at a 45° angle in relation to the collimated irradiation beam (Figure 1b), and Δ*t* = 180 min the total irradiation time.

### 2.5 Computational Methods

To understand the alanine-induced photodegradation of glycine (Section 3.2), we evaluated the photodegradation of glycine in a glycine-like environment versus in an alanine-like environment. To gain a qualitative understanding of this process, a bounding study using implicit solvation models and ground state density functional theory (DFT) was performed. Kohn–Sham Density Functional Theory (here denoted DFT) is an accurate, computationally affordable quantum mechanical method frequently used to predict the properties of molecules, bulk solids, and materials interfaces.^45^ Its computational affordability derives from the insight that the exact energy (i.e., the exact solution to the Schrodinger equation) could be determined from knowledge of the true electron density.^46^ It is a noted method of choice for calculating bond energies and barrier heights for main-group element containing molecules like amino acids;^47^ because of this, DFT was chosen to calculate the barriers for glycine and alanine decarboxylation and, in turn, to estimate rates of photodegradation.

Our initial bounding study revealed the effect that the polarity of the environment has on the kinetics of the simple decarboxylation of glycine. While this approach cannot capture the consequences of long-range structure or excited state effects, it does provide a structural explanation of the experimental observations. Future work involving condensed-phase models and excited states will also be valuable.

Using DFT via the ORCA^48–52^ quantum chemistry package, zwitterionic glycine was optimized using the B3LYP^53,54^ density functional, the def2-TZVP^55,56^ basis set, Grimme et al.’s D3 dispersion energy correction using Becke-Johnson damping,^57,58^ and the conductor-like polarizable continuum model^59^ (CPCM) for implicit solvation modeling. To model surrounding environments of differing polarity, the dielectric constant (ε) was adjusted while keeping all other CPCM parameters set to their default values; 15 values of ε within the 3–80 range were used (data tabulated in Table S3; example ORCA script in SI). The self-consistent field energy convergence threshold was tight at 10^–8^ Hartree. Frequency calculations verified that the geometric optimizations were to stationary minima and gave thermodynamic quantities. For every value of ε, the transition state for simple decarboxylation was also optimized. Frequency calculations obtaining exactly one imaginary carbon–carbon stretching mode and corresponding intrinsic reaction coordinate^60^ scans verified that the transition state optimizations correctly corresponded to simple decarboxylation. For a given value of ε, the Gibbs free energies of the transition state and the ground state were used to obtain the activation barriers for simple decarboxylation; using these barriers and the vibrational frequencies, harmonic transition state theory^61^ (HTST) was used to estimate reaction rates of simple decarboxylation from the glycine ground state (Tables 1 and S4; the key HTST equations are described in SI). Considering glycine and using key values of ε (4, 15, 18, 80), DFT functional and basis set benchmarking was performed using the def2-TZVPP basis set and the M06-L,^62^ M06,^63,64^ M06-2X,^63,64^ and ωB97X^65^ functionals. These benchmarking studies confirmed the overall conclusions obtained with the chosen DFT methodology (Table S4). The results for the water-like environment (ε of 80), agree with those found by Alexandrova and Jorgenson in their joint electronic structure and QM/MM Monte Carlo study.^66^ For key values of ε (4, 15, 18, 80), our DFT procedure was also performed for l-alanine (Table S5).

**Table 1:** Rates from Harmonic Transitions State Theory (HTST) and DFT at 298.15 K and 1 atm

ε	HTST effective frequency (cm^–1^)	Arrhenius prefactor (s^–1^)	Δ*G* ^‡^ (kcal mol^–1^)	rate (s^–1^)
4	11902.62	3.57 × 10^14^	43.4	5.31 × 10^–18^
10	10598.37	3.18 × 10^14^	46.1	4.94 × 10^–20^
15	10184.91	3.05 × 10^14^	46.8	1.47 × 10^–20^
18	10091.94	3.03 × 10^14^	47.0	9.72 × 10^–21^
20	10096.33	3.03 × 10^14^	47.1	7.99 × 10^–21^
30	9975.82	2.99 × 10^14^	47.5	4.27 × 10^–21^
80	9814.01	2.94 × 10^14^	48.0	1.91 × 10^–21^

### 2.6 Titan’s Atmospheric Radiative Fluxes

To calculate the expected solar irradiation in Titan’s summer pole mesosphere, we modeled the solar irradiation at 350 and 450 km, assuming the complete depletion of the organic aerosols above it. The summer pole irradiation was calculated using a zenith angle of 63.3°, given Titan’s obliquity of 26.7°. We used the temperature profile of Hörst^20^ and assumed the maintenance of trace molecules above the summer pole at the following mixing ratios: N_2_: 95%, CH_4_: 5%, hydrogen cyanide (HCN): 30 ppm, acetylene (C_2_H_2_): 30 ppm, ethylene (C_2_H_4_): 20 ppm, ethane (C_2_H_6_): 30 ppm, diacetylene (C_4_H_2_): 1 ppb, carbon dioxide (CO_2_): 10 ppb.^20^ The atmospheric radiative transfer was solved through a cloudless infinite-slab UV transport model that accounts for Rayleigh scattering. As described by Bangera et al.,^67^ we coupled the discrete ordinate radiative transfer code C-DISORT^68–71^ to ARGO.^67,72^ C-DISORT provides the exact solution of the plane-parallel radiative transfer equation for a given set of polar angles, denoted as streams. In the framework of a discrete ordinate radiative transfer scheme, the number of streams defines the numerical accuracy. In this work we employed four streams, usually enough for calculating angular-averaged quantities in the absence of strongly asymmetric scattering. The C-DISORT radiative transfer yields the mean intensity as a function of atmospheric height, which we subsequently converted to the actinic flux at the desired altitudes. Rayleigh scattering functions for CH_4_ and N_2_ were computed from the refractive indices of Sneep and Ubachs^73^ and Cox,^74^ respectively. The UV cross sections are those given in Rimmer et al.,^75^ taken from the PhiDRates,^76–78^ Mainz,^79,80^ and Leiden databases.^81–84^ Our model accounts for when cross sections were measured at different temperatures and for their temperature-dependences.

## 3 Results and Discussion

### 3.1 Irradiation of Pure Alanine and Glycine Samples

The irradiation of the pure glycine and pure alanine samples revealed very distinct behaviors (Figure 3). At our tested conditions, the photodegradation of the glycine nanolayer was barely distinguishable, with the degradation rate of its α-carbon functional group, measured at the ω­(CH_2_) band (1336 cm^–1^, Table S1), being close to zero—*J*_CH_2__^gly smp^ = (1.57 ± 1.38) × 10^–5^ min^–1^—as was its experimental quantum efficiency of degradation—Φ_exp_^gly smp^ = (1.7 ± 4.8) × 10^–4^ molecule photon^–1^. On the other hand, the photodegradation of alanine was significant. Its α-carbon δ­(CH) band (1306 cm^–1^, Table S1) degraded at a rate of *J*_CH_^ala smp^ = (21.51 ± 4.02) × 10^–5^ min^–1^, 1 order of magnitude higher than that of glycine. The alanine carboxylate group, monitored at the ν_as_(COO^–^) band (1588 cm^–1^), degraded at the rate of *J*_COO^–^_^ala smp^ = (29.22 ± 6.01) × 10^–5^ min^–1^, slightly faster than that measured at its α-carbon (Figure 3). Under these conditions, we measured a quantum efficiency of degradation of alanine of Φ_exp_^ala smp^ = (7.5 ± 10.1) × 10^–4^ molecule photon^–1^.

The measured photodegradation rates match well with similar irradiation experiments on thin layers of alanine—(14.34 ± 5.93) × 10^–5^ min^–1^—^30^ and glycine—(2.31 ± 0.51) × 10^–5^ min^–1^,^30^ (0.48 ± 1.74) × 10^–5^ min^–1^,^33^ (9.30 ± 8.40) × 10^–5^ min^–1^.^32^ Further, in previous studies, when both amino acids were irradiated under the same environmental conditions, alanine has consistently photodegraded faster than glycine upon ultraviolet irradiation.^30,35^ The faster degradation of alanine was justified by the higher stability of the alanine radical product—the ethylamine (^•^CHCH_3_NH_3_^+^) radical—compared to that of glycine—the methylamine (^•^CH_2_NH_3_^+^) radical—after the decarboxylation step,^30^ which initiates the UV-induced mineralization of the amino acids.^85,86^ Given the similarity of our experimental setup with that of ten Kate et al.,^30^ the same reasoning can account for the differences observed in our results from irradiation of the pure alanine and pure glycine samples (Figure 3).

### 3.2 Irradiation of Mixed Alanine and Glycine

During the irradiation of the alanine+glycine samples, the individual evolutions of alanine and glycine molecules were assessed based on their α-carbons IR bands—assigned to the δ­(CH) and ω­(CH_2_) modes of alanine and glycine, respectively (Figure 2). In the mixture sample, alanine degraded at the rate of *J*_CH_^ala+gly smp^ = (22.23 ± 1.47) × 10^–5^ min^–1^, unchanged relative to the pure alanine sample (Figure 4a). These results convey that the presence of glycine, at the 1:1 ratio, does not influence the photolysis of alanine (Figure 4b). This is consistent with the observations we made elsewhere^41^ where most properties of alanine are little influenced by glycine in the alanine+glycine sample (further discussed in Section 3.3). Instead, glycine is significantly influenced by the presence of alanine.^41^

Consistent with that, the presence of alanine changes significantly the photodegradation rate of glycine, increasing it 10-fold to *J*_CH_2__^ala+gly smp^ = (15.46 ± 3.46) × 10^–5^ min^–1^. The difference to the pure glycine sample is statistically significant—*t*(20) = 8.32, *p*_two‑tailed_ < 0.001 (Figure 4c). The same observation was made after correcting for the initial number of glycine molecules in each sample, with the glycine quantum efficiency of photodegradation in the alanine+glycine sample—Φ_exp, gly_^ala+gly smp^ = (5.7 ± 3.3) × 10^–4^ molecule photon^–1^—being also statistically higher than that in the pure glycine sample—*t*(4) = 2.99, *p*_two‑tailed_ = 0.040 (Figure 4c). These results demonstrate that the interaction of alanine with glycine has induced the latter to degrade under conditions under which it would otherwise remain inert.

### 3.3 The Role of α-Carbon Electron Density

As we previously determined, the electron density of the alanine and glycine α-carbons—different in their pure samples—became indistinguishable in the alanine+glycine sample.^41^ If the difference in electron density at the α-carbon accounts for the differing degradation rates between the two amino acids,^30^ then their common α-carbon electron density in the mixture sample^41^ should result in a comparable photodegradation rate for alanine and glycine. The degradation rate of glycine in the alanine+glycine sample was, however, consistently lower than the degradation rate of alanine—*t*(20) = 4.06, *p*_two‑tailed_ = 0.001—as reproduced in UV-enhanced irradiations experiments (Figure 5).

**Figure 5:**
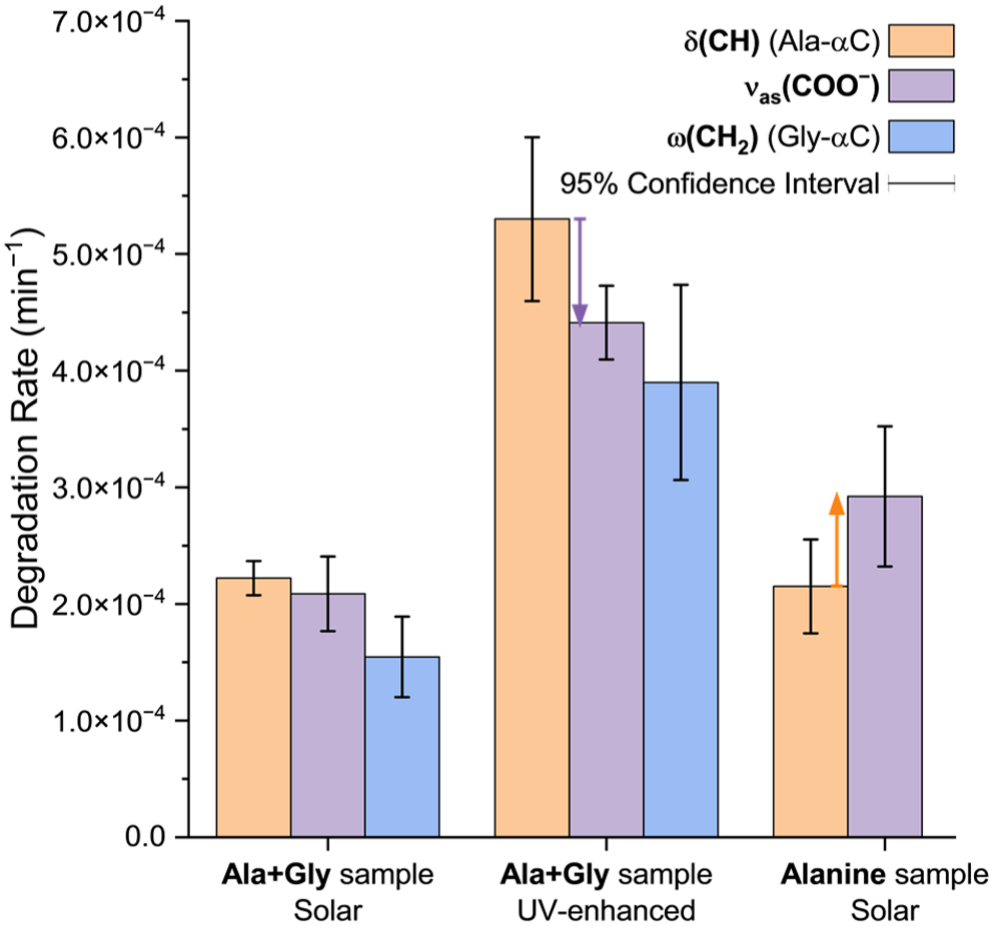
Degradation rates depending on sample composition and UV photon flux. In the pure alanine sample, the ν_as_(COO^–^) vibrational mode degrades faster than the δ­(CH) mode (orange arrow). Inversely, in the alanine+glycine sample, the ν_as_(COO^–^) mode degrades slower than the δ­(CH) mode. This trend is more pronounced when the alanine+glycine sample is irradiated with an UV-enhanced lamp (violet arrow).

Furthermore, whereas in the pure alanine sample the ν_as_(COO^–^) mode degraded slightly faster than the δ­(CH) mode—*t*(20) = 2.38, *p*_two‑tailed_ = 0.028—the opposite was true during the extended photodegradation of the mixture sample—*t*(20) = 2.57, *p*_two‑tailed_ = 0.018 (Figure 5). The fact that the −COO^–^ groups degraded more slowly than the alanine −CH– group in the alanine+glycine sample further supported the observation that glycine, the second contributor of carboxylate groups to the mixture sample, degraded more slowly than alanine when the amino acids were codeposited (Figure 5).

Our previous characterization showed that the alanine and glycine molecules attained common properties in the mixture sample.^41^ The properties of glycine were shown to be altered the most, having led to a marked difference in its degradation behavior (Figure 4c). Nevertheless, despite the equalization of the carboxylate- to α-carbon C–C bond polarity, the vibrational coupling of the shared alanine and glycine carboxylate and ammonium vibrational modes, and the homogeneity of the mixture nanolayer,^41^ alanine and glycine still degraded at different rates in the alanine+glycine sample. We therefore conclude that neither of the probed properties, particularly the α-carbon electron density, fully explains the difference in degradation rate between alanine and glycine. This conclusion is supported by the fact that the alanine degradation rate remained unchanged from the pure alanine to the alanine+glycine sample (Figure 4b), despite differences in its α-carbon electron density.^41^

### 3.4 The Role of Environment Polarity

We next asked whether the environment polarity felt by the amino acids could explain the difference in degradation rate between alanine and glycine and, consequently, the alanine-induced degradation of glycine. For that purpose, we compared the decarboxylation reaction rate of glycine in a glycine-like environment and an alanine-like environment.

A previous computational study^87^ estimated that alanine and glycine have ε values of roughly 15 and 18, respectively. As noted in that study, in implicit solvation models, water is modeled with an ε value of about 80, and protein environments are commonly modeled with a value of 4. With these key bounding values in mind, examine Figure 6. As the environment becomes more apolar (i.e., as ε decreases), Figure 6a,b respectively show monotonic decreases in the Gibbs free energy barrier for simple decarboxylation, Δ*G* ^**‡**^ (Figure 6a), and the length of the transition state’s intramolecular hydrogen bond (Figure 6b, bond shown as dashed line in the inset). Figure 6c shows that Δ*G* ^**‡**^ has a remarkably linear correlation with the hydrogen bond length (abbreviated as r_H‑Bond_ in the equation for the fit). Altogether, Figure 6 demonstrates that as the environment becomes more apolar, glycine attains a shorter intramolecular hydrogen bond, which consistently stabilizes its barrier for simple decarboxylation reaction. Simple decarboxylation rates were calculated using harmonic transition state theory and are presented in Table 1. Going from an ε value of 18 (placing glycine in a roughly approximate glycine-like environment) to 15 (placing glycine in a roughly approximate alanine-like environment), we measured a 1.5 times enhancement of the rate of simple decarboxylation. The trend is consistent with the experimental results. If we extend the examined range, we see a very high sensitivity to small changes in ε and, in turn, Δ*G* ^**‡**^. Going from an ε value of 30 to 10 (which extends the range in Δ*G* ^**‡**^ by only 1.2 kcal mol^–1^), which is similar to going between the averaged computationally determined values^87^ for the most and least polar amino acids, there is a ∼12 times rate enhancement.

**Figure 6:**
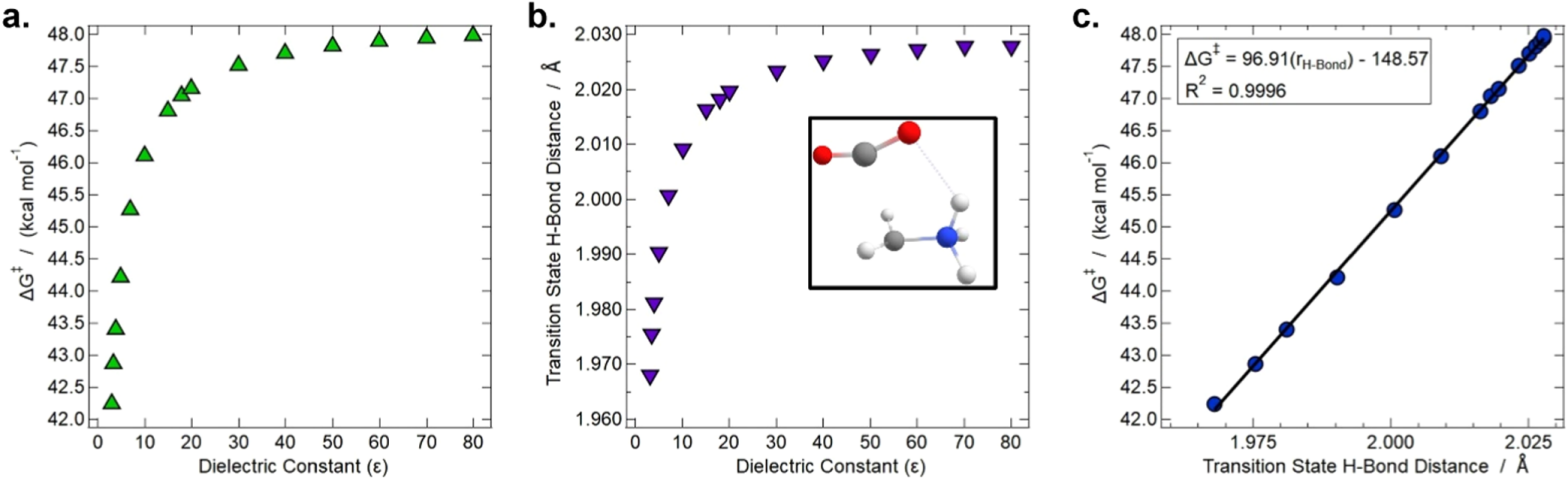
**a.** DFT calculated Gibbs free energy barrier height (Δ*G* ^‡^) for the glycine simple decarboxylation reaction versus ε. **b.** DFT optimized length of the simple decarboxylation transition state’s intramolecular hydrogen bond between the leaving CO_2_ molecule and remaining fragment (shown as the dashed bond in the inset image) versus ε. **c.** Δ*G* ^‡^ versus the transition state H-bond length.

The l-alanine rates for key values of epsilon are tabulated in Table S5. Consistent with the experimental results, alanine decarboxylation rates are equal in the alanine-like (ε of 15) and glycine-like (ε of 18) environments. The slow absolute rates for alanine may be due to the limitations of an implicit solvation model and the absence of intermolecular effects.

The DFT calculations allow us to conceptually connect the measured change in the α-carbon charge density^41^ to the experimental increase in photodegradation of glycine in an alanine-like environment. Hirshfeld^88^ atomic charges from the DFT calculations allow us to estimate the α-carbon charge computationally. In the Hirshfeld scheme, a portion of the calculated DFT electron density is attributed to each atom using weighting factors derived from considering an idealized atom in a “pro-molecule” made up of idealized atoms. The results reveal an environment polarity-driven intramolecular charge transfer within the transition state. Specifically, as the environment becomes more apolar (as ε decreases), the glycine intramolecular H-bond shortens, there is more electron density on the –^+^NH_3_ group at the expense of the fragmenting glycine, and, consequently, there is less electron density at the α-carbon (as indicated by the atomic charge value becoming more positive). This is seen in Figure 7, where, as the intramolecular hydrogen bond shortens (as glycine is placed in a more apolar environment), there is increased electron density on the –^+^NH_3_ group (Figure 7b) at the expense of the rest of the fragmented glycine, including the leaving CO_2_ group (Figure 7a), the α-carbon alone (Figure 7c), and the α-carbon plus the charge in its two bonded hydrogens (Figure 7d). Considering the α-carbon alone (Figure 7c), these results are consistent with the decreased electron density on the glycine α-carbon when it is codeposited in a 1:1 ratio with alanine.^41^ The changes in the charges within the functional groups are also remarkably linear with the transition state intramolecular hydrogen bond length.

**Figure 7:**
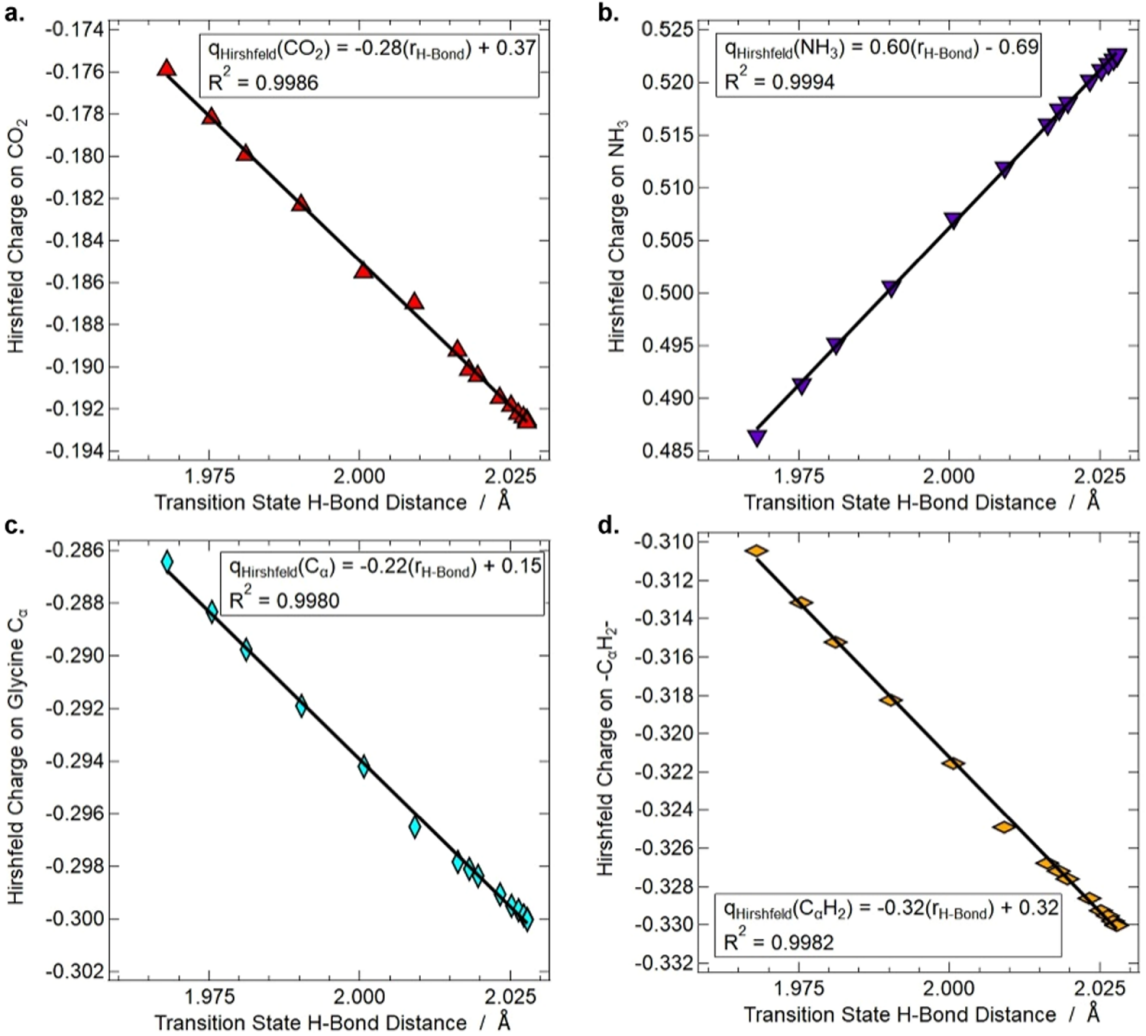
Hirshfeld charges, as a function of transition state hydrogen bond distance, on the CO_2_ group (**a**), the ^+^NH_3_ group (**b**), the α-carbon (**c**), and the α-carbon with its two attached hydrogens (**d**).

Overall, DFT calculations substantiate the faster degradation rate of glycine in the alanine+glycine sample compared to the pure glycine sample. The increased degradation is explained by the decreased polarity of an alanine-rich environment, which stabilizes the transition state of the glycine decarboxylation reaction. The DFT calculations also support the decrease in the glycine α-carbon electron density upon codeposition with alanine. Still, contrary to previous reasonings, this computational study suggests that the environment polarity—rather than the α-carbon electron density—explains the faster degradation rate of alanine compared to that of glycine and the alanine-induced degradation of glycine.

### 3.5 Implications to Titan’s Meridional Transportation

We extrapolated the laboratory amino acid degradation rates to Titan’s summer pole mesosphere. To model those conditions, we assumed the complete removal of the organic aerosols in the upper atmosphere, above the main haze deck and any existent detached haze layer.^89^ The following conclusions can be extended to all mesospheric altitudes, as the upper atmosphere is optically thin to mid-UV photons. The attenuation of the photon flux, above 200 nm, incident at the top of Titan’s atmosphere is negligible at an altitude of 450 km and exhibits only a 0.1% decrease at 350 km (Figure 8). As long as the organic molecules remain below altitudes at which their degradation is better explained by more energetic irradiation, the amino acids being transported through Titan’s summer pole mesosphere should be subject to the extrapolated photodegradation half-lives calculated in this study. The half-lives were extrapolated as described in Section 2.4. Each experiment simulated 2.21 Titan-day (35.2 Earth-day) of continuous irradiation of the summer pole.

**Figure 8:**
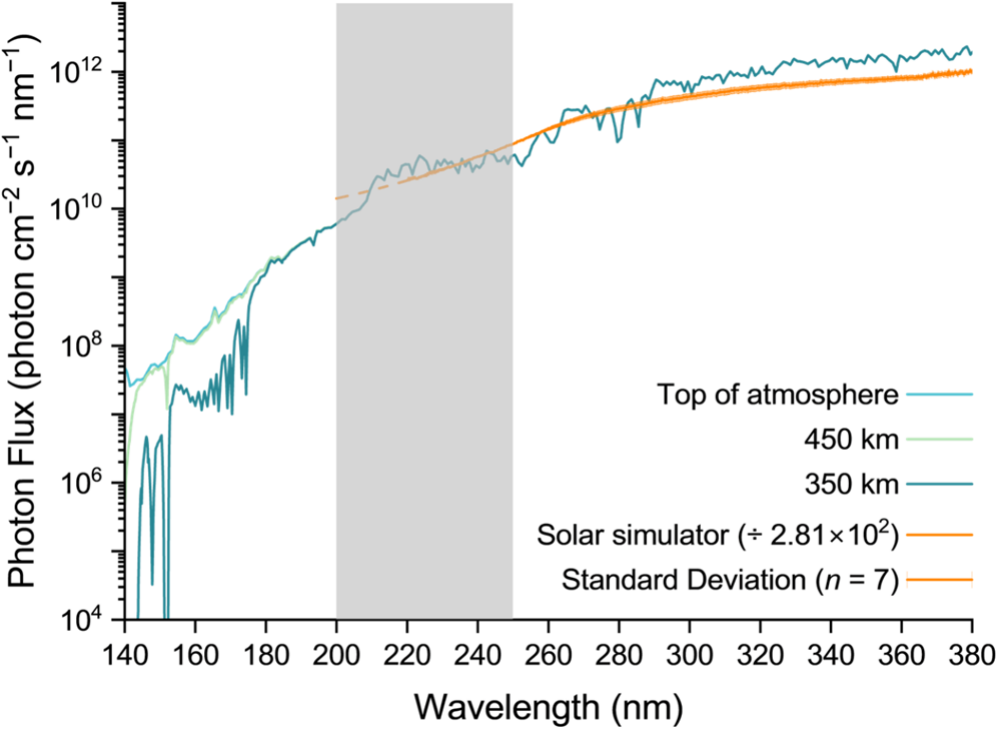
Modeled solar irradiation at 350 km, 450 km, and the top of the atmosphere of Titan, and solar-like irradiation spectrum employed in our experiments. Although we could not measure the photon flux of our solar simulator setup below 220 nm, we expect it to extend to 200 nm (dashed extrapolation). The photodegradation half-lives were extrapolated to Titan’s mesosphere using the photon flux in the 200–250 nm range as conversion factor (gray area).

In our simulation experiments, 4.2 ± 0.4% (±SE, *n* = 3) of the initial amount of alanine degraded in the pure alanine sample, yielding a half-life of 38.9 ± 8.5 Titan-day on Titan’s summer pole mesosphere (Table 2). The degradation extent of alanine remained unchanged when codeposited with glycine (4.3 ± 0.2%) or irradiated in the absence of the ice layer (3.7 ± 0.7%), corresponding to half-lives of 37.7 ± 3.6 and 34.4 ± 6.1 Titan-day, respectively.

**Table 2:** Alanine and Glycine Quantum Efficiency of Degradation (Φ_exp_), Destruction Cross-Section (σ_des_) Obtained from Our Experimental Work, and Extrapolated Degradation Rates (J) and Half-Lives (*t*_1/2_) to the Summer Pole Mesosphere of Titan^a^

				Titan’s summer pole mesosphere	
	amino acid	quantum efficiency of degradation, Φ_exp_ (molecule photon^–1^)	destruction cross-section, σ_des_ (cm^2^)	degradation rate, *J* (s^–1^)	half-life, *t*_1/2_ (Titan-day)
Samples with Ice Layer					
alanine	alanine	(7.5 ± 10.1) × 10^–4^	(6.8 ± 1.5) × 10^–21^	(1.3 ± 0.3) × 10^–8^	38.9 ± 8.5
glycine	glycine	(1.7 ± 4.8) × 10^–4^	(5.0 ± 4.5) × 10^–22^	(9.3 ± 8.4) × 10^–10^	535.0 ± 492.7
alanine+glycine	alanine	(8.4 ± 3.7) × 10^–4^	(7.1 ± 0.7) × 10^–21^	(1.3 ± 0.1) × 10^–8^	37.7 ± 3.6
	glycine	(5.7 ± 3.3) × 10^–4^	(4.9 ± 1.2) × 10^–21^	(9.2 ± 2.3) × 10^–9^	54.2 ± 13.8
Samples without Ice Layer					
alanine	alanine	(5.5 ± 0.9) × 10^–4^	(7.7 ± 1.4) × 10^–21^	(1.4 ± 0.3) × 10^–9^	34.4 ± 6.1
alanine+glycine	alanine	(8.6 ± 9.9) × 10^–4^	(7.8 ± 1.2) × 10^–21^	(1.5 ± 0.2) × 10^–9^	34.2 ± 5.4
	glycine	(7.5 ± 5.0) × 10^–4^	(7.6 ± 1.6) × 10^–21^	(1.4 ± 0.3) × 10^–9^	35.1 ± 7.6

a To compute the Φ_exp_, σ_des_ and the extrapolated *J* and *t*_1/2_ values, we considered the photon flux within the 200–250 nm range.

Given the photodegradation half-life of alanine, we may now compare it to the expected duration of its transport from Titan’s polar to equatorial regions. Unfortunately, the velocity of the meridional circulation on Titan remains to be measured directly.^90^ To mitigate that uncertainty, global circulation models (reviewed by Lebonnois et al.^91^) have been employed.^20^ Early models based on measurements obtained from the Voyager Infrared Interferometer Spectrometer (IRIS) instrument have estimated meridional velocities in the upper stratosphere of 5 cm s^–1^, consistent with more recent and sophisticated models.^91,92^ A similar velocity of 3 cm s^–1^ was estimated for the middle-atmosphere meridional winds from thermal emission spectra obtained by the Composite Infrared Radiometer–Spectrometer (CIRS) instrument onboard Cassini.^93^ Dynamical time scales of the meridional circulation are obtained by dividing the meridional scale (the distance equivalent of Titan’s radius) by the mean meridional wind velocity and are considered the time scale of interest in global circulations.^20,91^ An average meridional wind velocity of 4 cm^–1^ translates to a dynamical time scale of about 47 Titan-day, similar to the half-life of alanine. Since both photodegradation and transportation time scales are shorter than the duration of each pole-to-pole cell—0.5 Titan-yr ≈ 667 Titan-day—these processes predominantly determine the fate of the prebiotic molecules. The similarity of the time scales implies that half of the alanine molecules propelled into the mesosphere would survive their journey from the pole to the equator; with the remaining half being photodegraded.

Three factors may, however, increase the number of alanine molecules reaching the equator. First, despite the upper-atmosphere meridional wind velocities being largely unconstrained, they are modeled to reach up to 150 m s^–1^ in the thermosphere near 70° N.^94^ The existence of faster meridional winds at higher altitudes is consistent with the 100-fold increase in meridional wind velocity from the low troposphere (0.04 cm s^–1^) to the upper stratosphere (5.0 cm s^–1^).^95^ This suggests that meridional wind velocities in the mesosphere are faster than those herein considered, reducing the exposure time of the prebiotic molecules to the photodegrading solar irradiation, thus increasing their survivability. Second, the direct comparison between the meridional overturning time scale and the photodegradation rate assumes a constant photon flux throughout the meridional scale. However, we solely modeled the irradiation expected on the summer pole, the most irradiated latitude on Titan;^25,26^ the degradation half-life of alanine averaged over the meridional scale should therefore be higher than here calculated. Third, we assumed a complete depletion of the organic aerosols, even though a limited number of them remaining in the upper atmosphere should shield the transported molecules from a fraction of the solar irradiation. We have not accounted for that residual shielding, which will similarly increase the survivability of the organic molecules. Overall, despite the similarity in the degradation half-life of alanine to the meridional overturning time scale, it is likely that more than half of the alanine molecules embedded in water–ammonia icy aerosols survive the journey through Titan’s mesosphere from the summer pole to the equatorial regions.

This is particularly true for prebiotic molecules that are more photostable than alanine, such as glycine (Table 2).^30,35^ In our pure-glycine experiments, only 0.3 ± 0.2% of the glycine molecules degraded, translating into a 535.0 ± 492.7 Titan-day half-life in the summer pole mesosphere (Table 2). Therefore, glycine is very likely to survive the meridional transport toward the equator. A significantly different conclusion is drawn, however, if we consider its interaction with alanine. In our experiments, the presence of alanine decreased the half-life of glycine to 54.2 ± 13.8 Titan-day.

The 10-fold decrease in half-life is particularly relevant for an alternative aerosolization scenario suggested by Cordier et al.^11^ Regardless of being produced on the surface of cryovolcanic regions or generated in Titan’s deep interior and brought to the surface through icy crust convection,^11^ once prebiotic molecules are present on the surface they may be transported by runoff to Titan’s hydrocarbon seas. There, nitrogen-rich bubble streams^96^ may disperse them into the atmosphere embedded in hydrocarbon aerosols. Similar to the cryovolcanic regions, hydrocarbon seas are most common on the polar regions of Titan.^97,98^ We thus suggest that hydrocarbon aerosols embedding prebiotic molecules are subject to the same meridional transport explored so far. It is now important to note that alanine increased the photodegradation rate of glycine by lowering its environment polarity (Section 3.4). The significant change in the glycine photodegradation was due to a slight variation in dielectric constant from ε = 18, in a glycine-like environment, to ε = 15, in an alanine-like environment (Figure 6); the change should be much more significant if the dielectric constant is varied to ε = 1–2, as expected in methane-rich environments.^99^ Although an increased photodegradation rate of glycine in hydrocarbon aerosols lowers the possibility of this amino acid surviving the journey from a polar hydrocarbon sea to an equatorial location, it remains an unexpected behavior whose consequences are worthy of further exploration.

## 4 Conclusions

We evaluated a possible transportation mechanism for amino acids produced in the polar cryovolcanic regions of Titan to reach its equatorial regions. Doing so, we constrained the likelihood of NASA’s Dragonfly lander sampling cryo-volcanogenic alanine and glycine. The amino acids are produced from the hydrolysis of organic macromolecules in cryolava flows.^2,4–7^ Building on previous suggestions,^11^ the exsolution of gases from the cryolava flows may then propel them into the atmosphere embedded in water–ammonia icy aerosols. We propose that these aerosols can hitchhike the pole-to-pole Hadley circulation, taking them from the summer pole to the equator.

The major impediment to this transportation occurs in the top branch of the Hadley circulation, where the prebiotic molecules will be subject to energetic solar ultraviolet irradiation, most significantly in the summer pole.^25,26^ We investigated the photodegradation half-life of alanine and glycine at these conditions, when embedded in a cryovolcanic environment.

At present knowledge, a sufficient fraction of alanine and glycine molecules are likely to survive the journey. Nevertheless, the photodegradation half-life of alanine—and glycine in the presence of alanine—is within the same order of magnitude as the meridional circulation time scale. This raises some uncertainty which merits further study. The largest uncertainty lies in the meridional transportation time scales in the mesosphere. To help constrain these, dedicated missions^90^ and instruments^100^ would be valuable. With better constrained degradation half-lives and circulation time scales, we can more plausibility conclude whether Dragonfly can detect cryovolcanic aerosols sedimented in the equatorial dune fields and interdune regions. Their identification could be performed by the Dragonfly Mass Spectrometer,^101^ when the detection of prebiotic molecules within the organic sand^102^ would correlate with high water and ammonia concentrations.

Lastly, we observed an unexpected 10-fold increase in the photodegradation rate of glycine when irradiated in the presence of alanine. Consistent with previous reasonings by ten ten Kate et al.,^33^ we have correlated the increased degradation of glycine with variations in the glycine α-carbon electron density.^41^ However, the latter did not predict the results obtained from the irradiation of the amino acid mixture. Backed by computational studies, we suggest that the lower polarity of an alanine-rich environment stabilized the glycine decarboxylation transition state and thus increased its degradation rate. This unexpected behavior has significant consequences for astrochemistry and the planetary sciences. It teaches us that by exclusively considering the effects of inorganic surfaces, we disregard a significant share of the photochemical fates of organic molecules. Further research exploring the effect of organic interactions^41^ in the photochemistry of prebiotic molecules is needed.

## Supplementary Material



## Data Availability

The data underlying the irradiation experiments are openly available in Figshare at dx.doi.org/10.6084/m9.figshare.28485524.

## Notes

Characterization of the amino acid and ice layers, irradiation spectra and list of experiments, and additional DFT information and results (PDF)
