# Supplementary material for: The Photochemistry of Amino Acids Produced on the Polar Cryovolcanic Regions of Titan
Source: ACS Earth Space Chem. 2025 Mar 8;9(3):715–28. doi: 10.1021/acsearthspacechem.4c00376 (PMC12818833; doi:10.1021/acsearthspacechem.4c00376)
Supplement: Supplementary file 1 [file sp4c00376_si_001.pdf]

The photochemistry of amino acids produced on the polar cryovolcanic regions of Titan

Diogo Gonçalves[1,2,†], Florence Hofmann[3,†], Severin Wipf[3], Riccardo G. Urso[4], Jana Bocková[5], Cornelia Meinert[5], Paul B. Rimmer[6], Gautam D. Stroscio[7], Nir Goldman[7,8], Andreas Elsaesser[3], Bruno Pedras[2,9,‡], Zita Martins[1,‡,*]

[1]Centro de Química Estrutural, Institute of Molecular Sciences and Department of Chemical Engineering, Instituto Superior Técnico, Universidade de Lisboa, Av. Rovisco Pais 1, 1049-001 Lisbon, Portugal

[2]Institute for Bioengineering and Biosciences and Department of Chemical Engineering, Instituto Superior Técnico, Universidade de Lisboa, Av. Rovisco Pais 1, 1049-001 Lisbon, Portugal

[3]Freie Universität Berlin, Department of Physics, Experimental Biophysics and Space Science, Arnimallee 14, 14195 Berlin, Germany

[4]INAF-Osservatorio Astrofisico di Catania, Via Santa Sofia 78, 95123 Catania, Italy

[5]Université Côte d'Azur-CNRS, ICN, UMR7272, 06108 Nice, France

[6]Cavendish Laboratory, University of Cambridge, JJ Thomson Ave, CB3 0HE Cambridge, UK

[7]Lawrence Livermore National Laboratory, Livermore, California 94550, United States

[8]Department of Chemical Engineering, University of California, Davis, California 95616, United States

[9]Associate Laboratory i4HB—Institute for Health and Bioeconomy, Instituto Superior Técnico, Universidade de Lisboa, Av. Rovisco Pais 1, 1049-001 Lisbon, Portugal

†These authors contributed equally to this work

‡These authors share senior authorship

*Corresponding author: zita.martins@tecnico.ulisboa.pt

Table of contents

FT-IR characterization of the amino acid nanolayers 3

Ice layer characterization 4

The ice layer influence on the amino acid photodegradation 4

Irradiation spectra 6

Irradiation experiments 7

DFT suplementary information 8

Key equations for harmonic transition state theory 8

Example ORCA input script 8

References 11

FT-IR characterization of the amino acid nanolayers

Table S1. Assignment of the distinguishable bands of the alanine+glycine sample infrared spectrum, collected at room temperature, and their equivalents in the pure amino acid (aa) samples.

Assignment*	**Alanine+Glycine** $\bar{\nu}$ (cm^{-1})	Equivalent band in the pure aa samples<br>**Alanine** $\bar{\nu}$ (cm^{-1})	 **Glycine** $\bar{\nu}$ (cm^{-1})
$\rho(^{+}NH_3)$	1014	1012	-
$\nu_{as}(CCN)$	1042	-	1040
$\rho(^{+}NH_3)$	1116	1110	1118, 1136
$\rho(CH)$	1238	1230	-
$\delta(CH)$	1308	1306	-
$\omega(CH_2)$	1330	-	1336
$\delta_s(CH_3)$	1360	1356, 1366	-
$\nu_s(COO^-)$	1412	1412	1414
$\delta(CH_2)$	1442	-	1446
$\delta_{as}(CH_3)$	1450	1454[c]	-
$\delta_s(^{+}NH_3)$	1522	1522[c]	1522
$\nu_{as}(COO^-)$	1594	1588	1602
$\delta_{as}(^{+}NH_3) + \tau(^{+}NH_3)$	2116	2108	2134
$\nu_s(^{+}NH_3)$, $\nu_s(C\text{-}H)$	2530–3000	2530–3000	2530–3000
$\nu_{as}(^{+}NH_3)$	3076	3044	3180

*ρ, rocking; ν, bond stretching; δ, scissoring; ω, wagging; τ, torsion; as, antisymmetric; s, symmetric.

Ice layer characterization

In the mid-IR spectra acquired at 90 K, the main absorption feature of water and ammonia overlap.[1–3] However, the ammonia ν_2 umbrella mode at 1105 cm^{-1} does not overlap with any water absorption band,[1,2] allowing us to quantify the ammonia present in the ice layer (Figure S1). For that, we assumed a band strength for the ν_2 ammonia band[1] of A = 1.4 × 10^{-17} cm molecule^{-1} and A = 2.0 × 10^{-16} cm molecule^{-1} for the ν_1 water band[4] at 3256 cm^{-1}. From the integrated absorption of the latter we subtracted the contribution from the overlapping ν_3 ammonia band—estimated from the integrated ν_2 ammonia band absorption—assuming a band strength of A = 1.8 × 10^{-17} cm molecule^{-1}.[1] We thus computed a column density of N = 2.3 × 10^{16} molecule cm^{-2} for ammonia and N = 5.8 × 10^{17} molecule cm^{-2} for water, translating into a water:ammonia ratio of 96:4, close to the desired 95:5 ratio. The difference to the desired ratio may be explained by the proximity to the ammonia sublimation temperature, which could reduce the amount of ammonia trapped in the water ice. Besides, the concentration of ammonia in the ice layer may be higher than measured, as the intensity of the ammonia umbrella band is attenuated in water-dominated ices, becoming up to 1.3 times smaller than in the pure ammonia ice[5] in which was measured the absorption coefficient of the ν_2 mode.[1] As the ammonia ν_2 vibrational mode overlaps with some of the alanine and glycine modes, we considered only the ν_1 water mode to quantify the ice layer thickness (assuming a 100% water ice), having obtained a mean ice thickness of 217 ± 39 nm (± *SD*, *n* = 21) throughout our experiments.

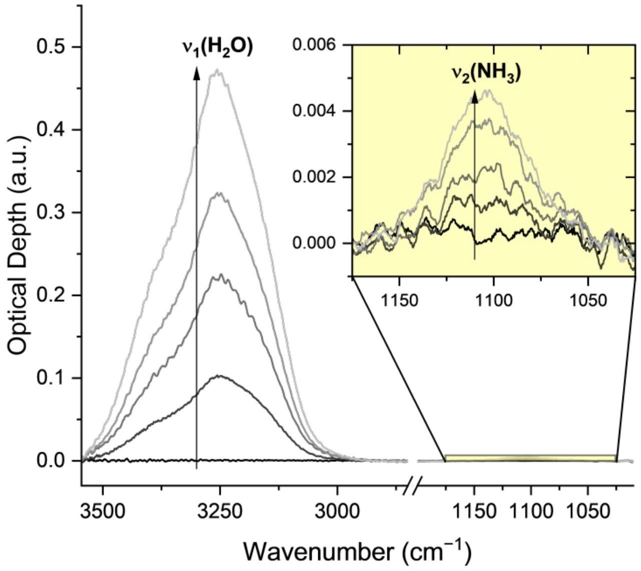

Figure S1. Evolution of the bulk O-H stretching band of water—$\nu_1(H_2O)$—and the ammonia umbrella band—$\nu_2(NH_3)$—during the vacuum deposition of the water-ammonia mixture on an empty CaF_2 window.

The ice layer influence on the amino acid photodegradation

Since early astrochemistry experiments, the effect of ice on the degradation of amino acids and other organic molecules has been considered by embedding them in ice matrices.[6–10] When the effect of the ice matrix on the ultraviolet photolysis of amino acids was isolated,[6] the ice matrix was shown to slightly extend the molecule lifetime, consistent with similar recent experiments.[11] This influence was suggested to become negligible,

however, in optically thin ice layers.[6] Likewise, recent experiments with other prebiotically relevant molecules trapped in ice matrices, irradiated within the vacuum-UV range (120–180 nm), agree that the main effect of the water ice on the photodegradation of the organic molecules is the absorption of the ultraviolet radiation, merely decreasing the photon flux that reaches the organic molecules, without any interference in their inherent destruction cross sections.[12–14] This is consistent with the unchanged amino acid vibrational modes during the growth of the water-ammonia ice layer on top of our amino acid nanolayers (Figure S2). Attempts to test the influence of the ice layer on the photodegradation rate of the amino acids were inconclusive. Nevertheless, given our irradiation energies lower than 200 nm, we do not expect any photon absorption or radical formation within the thin ice layers[15] interfering with the photodegradation of the amino acids.

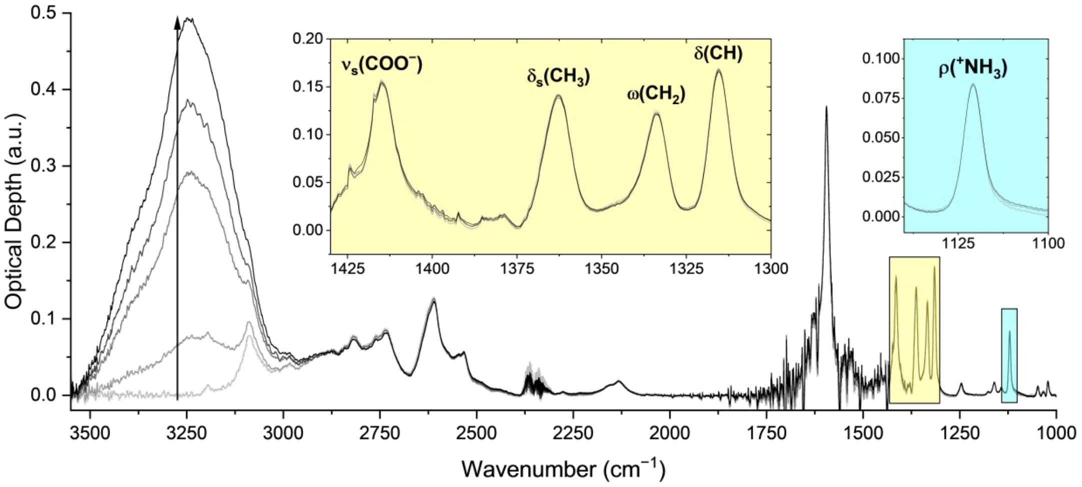

Figure S2. Time evolution of the alanine+glycine sample transmission FT-IR spectrum during the deposition of the water-ammonia ice layer. All bands of the deprotonated and protonated (-COO^- and -$^+NH_3$) as well as molecule-specific (-CH_2-, -CH-, and -CH_3) functional groups remain unaltered.

Irradiation spectra

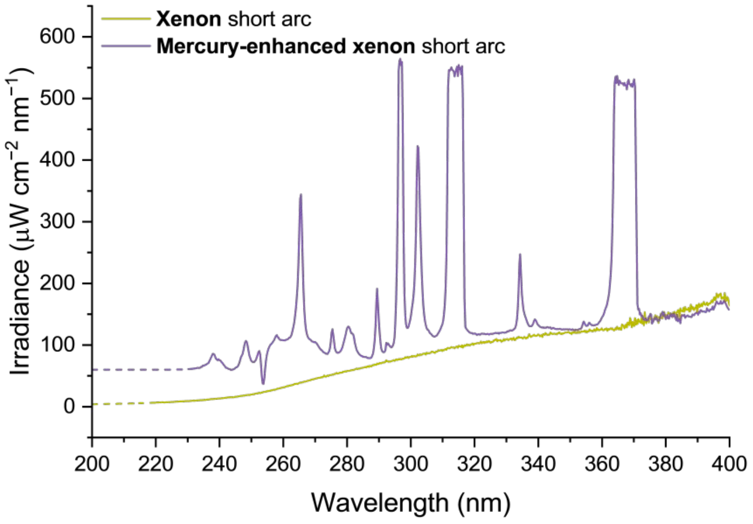

Figure S3. Irradiation spectra of the xenon and mercury-enhanced xenon short arc irradiation lamps employed in this work. Due to experimental limitations, their photon fluxes at low wavelengths were computed from the extrapolation (dashed) of the spectra down to the expected cut-off at 200 nm.

Irradiation experiments

Table S2. List of irradiation experiments performed in this work, combining different amino acid nanolayers with the presence of the water-ammonia ice layer and with one of two irradiation lamps.

Amino acid composition	Deposition of water-ammonia ice layer	Irradiation lamp
alanine	Yes	Xenon short arc
glycine	Yes	Xenon short arc
alanine+glycine	Yes	Xenon short arc
alanine	No	Xenon short arc
alanine+glycine	No	Xenon short arc
alanine+glycine	Yes	Mercury-enhanced xenon short arc

DFT supplementary information

Key equations for harmonic transition state theory[16]

$$\Delta G^{\ddagger} = G_{TS} - G_{GS} \quad (S1)$$

$$A = \frac{\prod_{i=1}^{3N-6} \nu_{i,GS}}{\prod_{i=1}^{3N-7} \nu_{i,TS}} \quad (S2)$$

$$k = A \exp\left(\frac{-\Delta G^{\ddagger}}{RT}\right) \quad (S3)$$

G_{TS} and G_{GS} are the Gibbs free energies of the transition state and the ground state, respectively. $\nu_{i,TS}$ and $\nu_{i,GS}$ is the ith vibrational mode frequency for the transition state and the ground state, respectively. R is the ideal gas constant (the molar equivalent of the Boltzmann constant), 8.31 J mol^{-1} K^{-1}. Rates were calculated at 298.15 K and 1 atm.

Example ORCA input script

```
! RKS B3LYP/G def2-TZVP D3BJ def2/J OptTS FREQ RIJCOSX TightSCF CPCM DEFGRID3
! NormalPrint PrintBasis PrintMOs

%pal nprocs 8
end
%maxcore 7000

%geom
Calc_Hess true
end


%cpcm
epsilon 18
end

%output
Print[ P_Hirshfeld ] 1
end

*xyz 0 1
[xyz coordinates here]
*
```

Table S3. Tabulated values from glycine DFT investigations.

**ε**	**G_{GS} (Hartree)**	**G_{TS} (Hartree)**	**ΔG‡ (kcal mol⁻¹)**	**TS H-Bond Length (Å)**	**TS COO^- Hirshfeld Charge**	**TS $^+NH_3$ Hirshfeld Charge**	**TS C_α Hirshfeld Charge**	**TS $C_\alpha H_2$ Hirshfeld Charge**
3	−284.51798001	−284.45066828	42.2	1.96806	−0.175929	0.486413	−0.286448	−0.310477
3.5	−284.52034438	−284.45202965	42.9	1.97549	−0.178211	0.491364	−0.288337	−0.313146
4	−284.52223027	−284.45307276	43.4	1.98115	−0.179939	0.495148	−0.28977	−0.315205
5	−284.52503432	−284.45456915	44.2	1.99027	−0.182352	0.500622	−0.291907	−0.318266
7	−284.52846022	−284.45632787	45.3	2.00066	−0.185544	0.507091	−0.294185	−0.321543
10	−284.53114900	−284.45768607	46.1	2.00917	−0.186982	0.511901	−0.296513	−0.324915
15	−284.53333183	−284.45876079	46.8	2.01631	−0.189234	0.516022	−0.297839	−0.326785
18	−284.53407739	−284.45912743	47.0	2.01823	−0.190182	0.517393	−0.2981	−0.327208
20	−284.53444706	−284.45931122	47.1	2.01963	−0.19045	0.51806	−0.298367	−0.327607
30	−284.53557975	−284.45986381	47.5	2.02334	−0.191517	0.520129	−0.299056	−0.32861
40	−284.53615337	−284.46014212	47.7	2.02519	−0.191884	0.521142	−0.299501	−0.329256
50	−284.53649884	−284.46030843	47.8	2.02635	−0.192269	0.521777	−0.299679	−0.329505
60	−284.53672973	−284.46042176	47.9	2.02724	−0.1924	0.522203	−0.299875	−0.3298
70	−284.53689492	−284.46050193	47.9	2.02780	−0.192538	0.522502	−0.299986	−0.329961
80	−284.53701912	−284.46055981	48.0	2.02787	−0.192661	0.522682	−0.30004	−0.33002

Table S4. Results from glycine DFT benchmarking investigations.

**ε**	**Material**	**M06-L/ def2-TZVPP ΔG‡ (kcal mol⁻¹)**	**M06/ def2-TZVPP ΔG‡ (kcal mol⁻¹)**	**B3LYP-D3(BJ)/ def2-TZVP ΔG‡ (kcal mol⁻¹)**	**M06-2X/ def2-TZVPP ΔG‡ (kcal mol⁻¹)**	**ωB97X/ def2-TZVPP ΔG‡ (kcal mol⁻¹)**
4	protein	38.2	40.7	43.4	47.3	49.3
15	alanine	41.7	44.4	46.8	50.8	53.2
18	glycine	42.0	44.6	47.0	51.0	53.4
80	water	42.9	45.6	48.0	52.0	54.5

Table S5. Results from alanine DFT investigations.

ε	HTST Effective Frequency (cm^{-1})	Arrhenius Prefactor (s^{-1})	ΔG‡ (kcal mol^{-1})	Rate (s^{-1})
4	14454.59	4.33 x 10^{14}	49.4	2.67 x 10^{-22}
15	5708.65	1.71 x 10^{14}	53.0	2.14 x 10^{-25}
18	7221.87	2.17 x 10^{14}	53.2	2.06 x 10^{-25}
80	14733.59	4.42 x 10^{14}	54.0	1.19 x 10^{-25}

References

(1) Zanchet, A.; Rodríguez-Lazcano, Y.; Gálvez, Ó.; Herrero, V. J.; Escribano, R.; Maté, B. Optical Constants of NH3 and NH3:N2 Amorphous Ices in the near-Infrared and Mid-Infrared Regions. *Astrophys J* **2013**, *777* (1), 26. https://doi.org/10.1088/0004-637X/777/1/26.

(2) Zheng, W.; Jewitt, D.; Kaiser, R. I. Infrared Spectra of Ammonia-Water Ices. *Astrophys J Suppl Ser* **2009**, *181* (1), 53. https://doi.org/10.1088/0067-0049/181/1/53.

(3) Moore, M. H.; Ferrante, R. F.; Hudson, R. L.; Stone, J. N. Ammonia–Water Ice Laboratory Studies Relevant to Outer Solar System Surfaces. *Icarus* **2007**, *190* (1), 260–273. https://doi.org/10.1016/J.ICARUS.2007.02.020.

(4) Gerakines, P. A.; Schutte, W. A.; Greenberg, J. M.; van Dishoeck, E. F.; Gerakines, P. A.; Schutte, W. A.; Greenberg, J. M.; van Dishoeck, E. F. The Infrared Band Strengths of H2O, CO and CO2 in Laboratory Simulations of Astrophysical Ice Mixtures. *Astron Astrophys* **1995**, *296*, 810. https://doi.org/10.48550/ARXIV.ASTRO-PH/9409076.

(5) Kerkhof, O.; Schutte, W. A.; Ehrenfreund, P. The Infrared Band Strengths of CH3OH, NH3 and CH4 in Laboratory Simulations of Astrophysical Ice Mixtures. *Astron Astrophys* **1999**, *346*, 990–994.

(6) Ehrenfreund, P.; Bernstein, M. P.; Dworkin, J. P.; Sandford, S. A.; Allamandola, L. J. The Photostability of Amino Acids in Space. *Astrophys J* **2001**, *550* (1), L95–L99. https://doi.org/10.1086/319491.

(7) Orzechowska, G. E.; Goguen, J. D.; Johnson, P. V.; Tsapin, A.; Kanik, I. Ultraviolet Photolysis of Amino Acids in a 100 K Water Ice Matrix: Application to the Outer Solar System Bodies. *Icarus* **2007**, *187* (2), 584–591. https://doi.org/10.1016/J.ICARUS.2006.10.018.

(8) Gerakines, P. A.; Hudson, R. L.; Moore, M. H.; Bell, J. L. In Situ Measurements of the Radiation Stability of Amino Acids at 15–140 K. *Icarus* **2012**, *220* (2), 647–659. https://doi.org/10.1016/J.ICARUS.2012.06.001.

(9) Materese, C. K.; Gerakines, P. A.; Hudson, R. L. The Radiation Stability of Thymine in Solid H2O. *Astrobiology* **2020**, *20* (8), 956–963. https://doi.org/10.1089/AST.2019.2199.

(10) Urso, R. G.; Scirè, C.; Baratta, G. A.; Brucato, J. R.; Compagnini, G.; Kaňuchová, Z.; Palumbo, M. E.; Strazzulla, G. Infrared Study on the Thermal Evolution of Solid State Formamide. *Physical Chemistry Chemical Physics* **2017**, *19* (32), 21759–21768. https://doi.org/10.1039/C7CP03959J.

(11) Dalla Pria, G. L.; Sohier, O.; Scirè, C.; Urso, R. G.; Baratta, G. A.; Palumbo, M. E. Experimental Study on the Radiation-Induced Destruction of Organic Compounds on the Surface of the Moon. *Icarus* **2024**, *415*, 116077. https://doi.org/10.1016/J.ICARUS.2024.116077.

(12) Maté, B.; Molpeceres, G.; Tanarro, I.; Peláez, R. J.; Guillemin, J. C.; Cernicharo, J.; Herrero, V. J. Stability of CH3NCO in Astronomical Ices under Energetic Processing. A Laboratory Study. *Astrophys J* **2018**, *861* (1), 61. https://doi.org/10.3847/1538-4357/AAC826.

(13) Maté, B.; Carrasco-Herrera, R.; Timón, V.; Tanarro, I.; Herrero, V. J.; Carrascosa, H.; Caro, G. M. M.; González-Díaz, C.; Jiménez-Serra, I. 2-Aminooxazole in Astrophysical Environments: IR Spectra and Destruction Cross Sections for Energetic Processing. *Astrophys J* **2021**, *909* (2), 123. https://doi.org/10.3847/1538-4357/ABDC1F.

(14) Herrero, V. J.; Tanarro, I.; Jimenez-Serra, I.; Carrascosa, H.; Muñoz Caro, G. M.; Mate, B. Stability of Urea in Astrophysical Ices. A Laboratory Study of VUV Irradiation and High-Energy Electron Bombardment. *Mon Not R Astron Soc* **2022**, *517* (1), 1058–1070. https://doi.org/10.1093/MNRAS/STAC2658.

(15) ten Kate, I. L.; Garry, J. R. C.; Peeters, Z.; Foing, B.; Ehrenfreund, P. The Effects of Martian near Surface Conditions on the Photochemistry of Amino Acids. *Planet Space Sci* **2006**, *54* (3), 296–302. https://doi.org/10.1016/J.PSS.2005.12.002.

(16) Vineyard, G. H. Frequency Factors and Isotope Effects in Solid State Rate Processes. *Journal of Physics and Chemistry of Solids* **1957**, *3* (1–2), 121–127. https://doi.org/10.1016/0022-3697(57)90059-8.
